# Supplementary material for: Molecular signatures and causal factors underlying latent cytomegalovirus infection among people living with HIV (PLHIV)
Source: Nat Commun. 2026 Mar 25;17:2871. doi: 10.1038/s41467-026-70889-z (PMC13022287; doi:10.1038/s41467-026-70889-z)
Supplement: Supplementary file 1 — Supplementary Information [file 41467_2026_70889_MOESM1_ESM.pdf]

Supplementary Info

Supplementary Figures

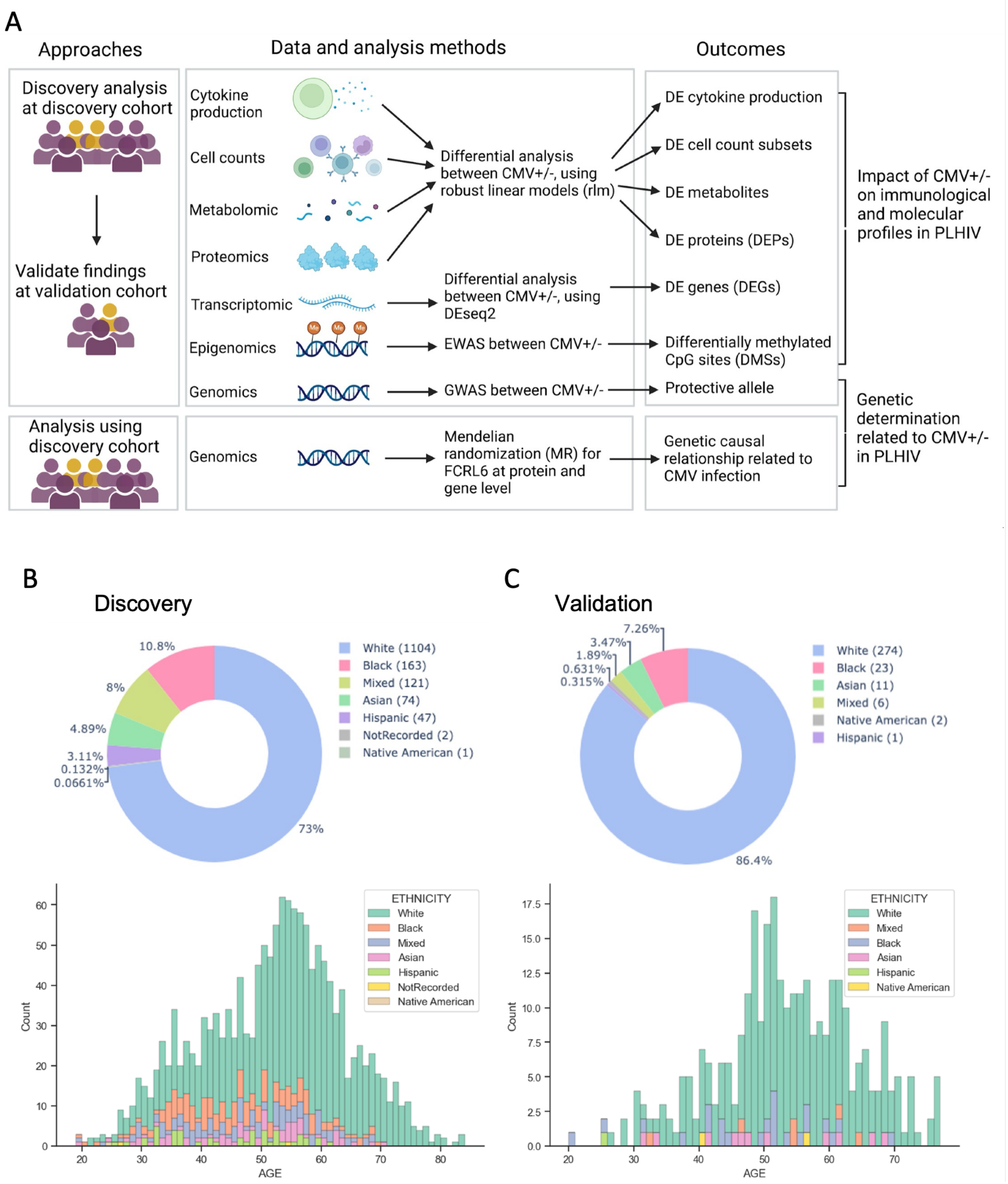

Supplementary Figure S1. Study design and cohort overview

A. Study design. Created in BioRender. Gödecke, S. (2025) https://BioRender.com/dz2rpem

B. Ethnicity percentage with age distribution in the Discovery cohort

C. Ethnicity percentage with age distribution in the Validation cohort

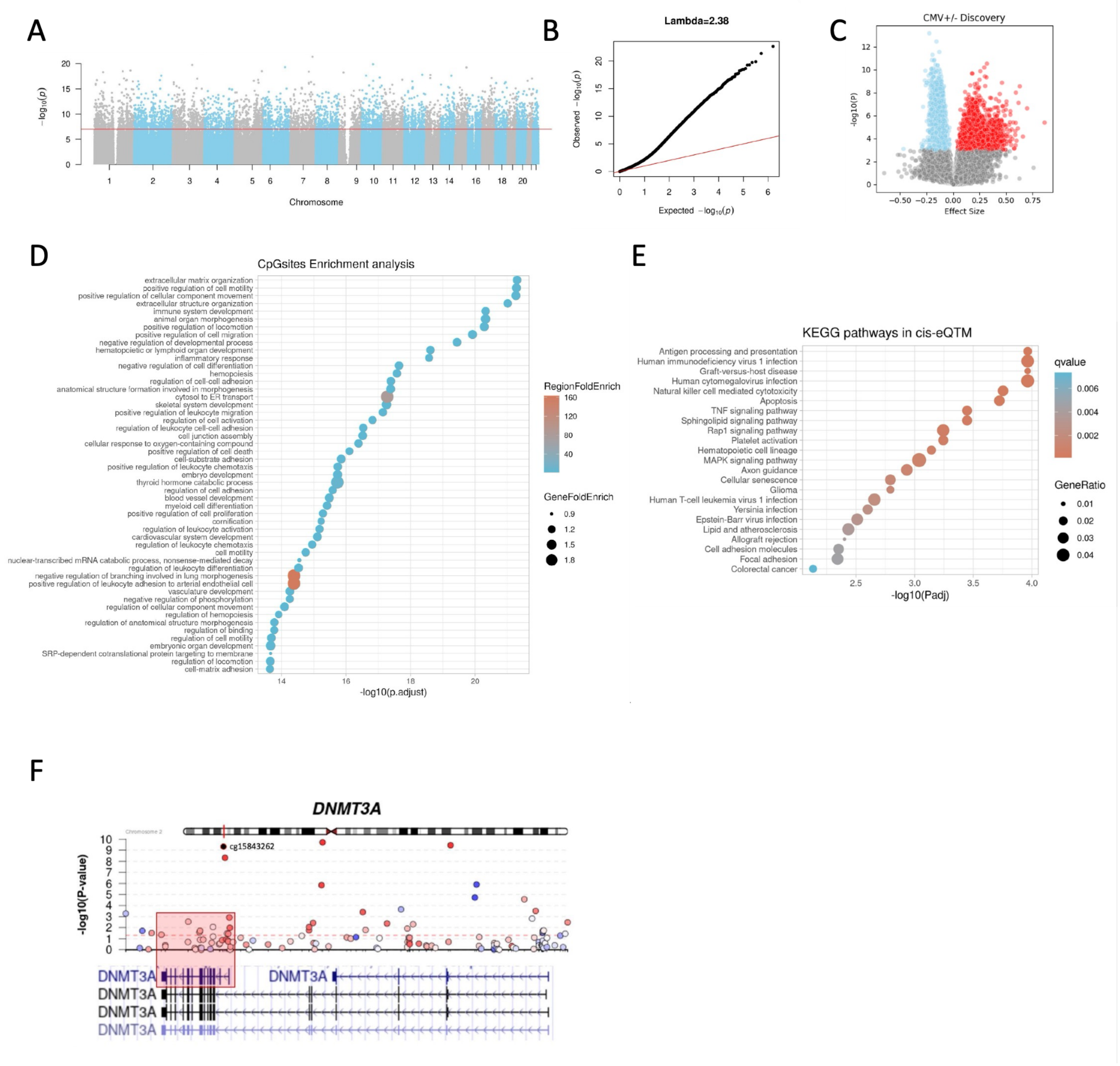

Supplementary Figure S2. Methylation analysis

A. Manhattan plot of EWAS results without BACON adjustment. P-values are from robust linear models adjusted for covariates.

B. QQplot of the raw p-value extracted from EWAS results.

C. Volcano plot of p-value and DNA methylation alteration. Red: increased methylated. Blue: decreased methylated. P-values and effect sizes are from robust linear models adjusted for covariates.

D. CpG sites Enrichment analysis. Associated genes are obtained based on GREAT version 4.0.4 with default settings.

E. GO Biological Processes enrichment of the cis-eQTM significant genes. FDR<0.05.

F. DNA methylation change of CpG site in the *DNMT3A* region. Cg15843262 is used as the reference. Red box: a DNMT3A transcript influenced by cg15843262. P-values are from robust linear models adjusted for covariates.

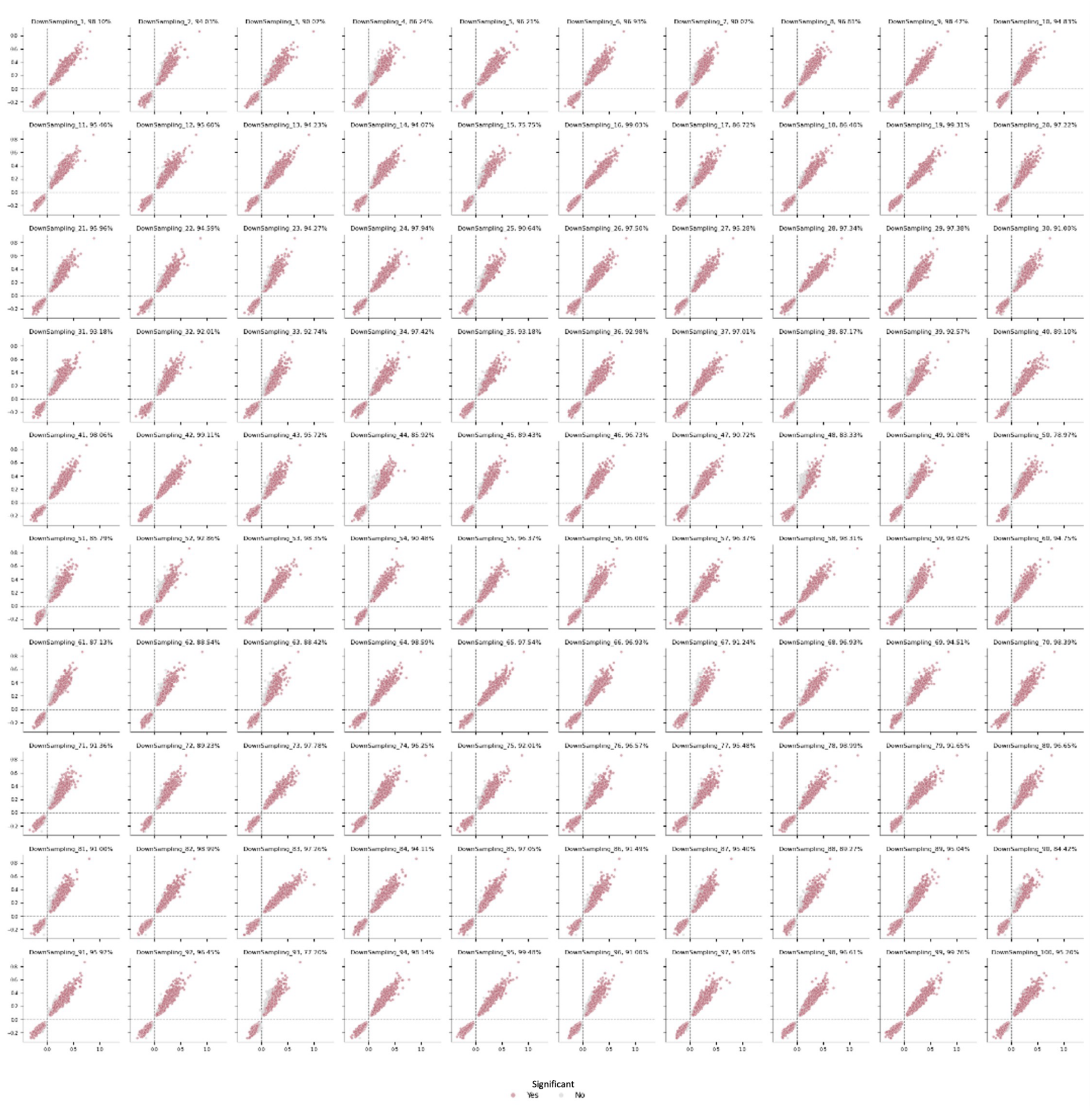

Supplementary Figure S3. Methylation downsampling analysis.

Correlation between effect sizes from the full DNA methylation analysis (x-axis) and the downsampled analyses (y-axis). The darker colour indicates if the CpG site was also significant in the respective downsampling analysis (FDR < 0.05). The percentages of significant CpG sites (FDR < 0.05) replicated in the downsampling iterations are in the range between 85% - 98%.

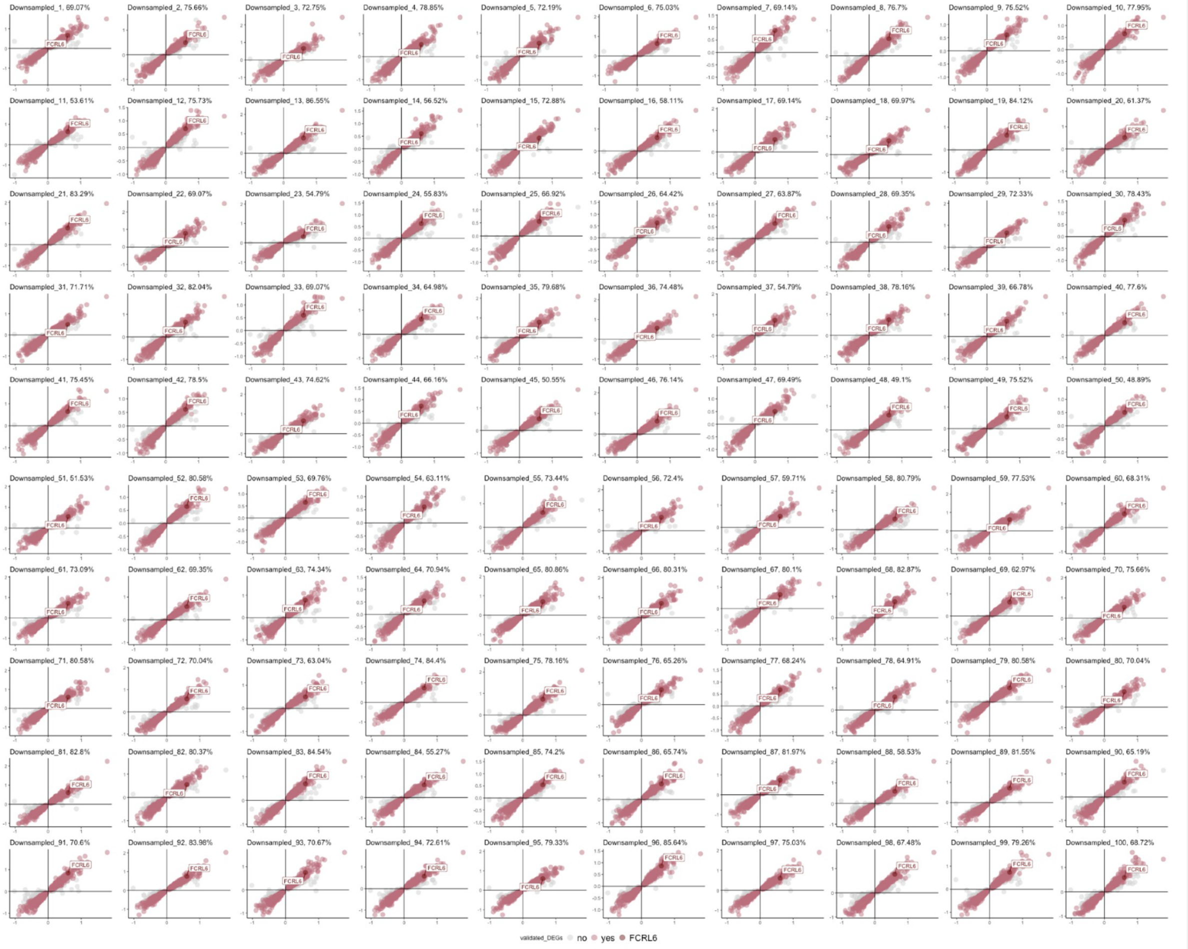

Supplementary Figure S4. Gene expression downsampling analysis.

The concordance plots for re-performing the bulk RNAseq analysis across 100 downsampling iterations in the discovery cohort. The percentages of significant genes (FDR < 0.05) replicated in the downsampling iterations are in the range between 48.89% - 86.55% with mean = 71.49%. The x-axis is the effect size from original analysis. The y-axis is the effect size from downsampling analysis.

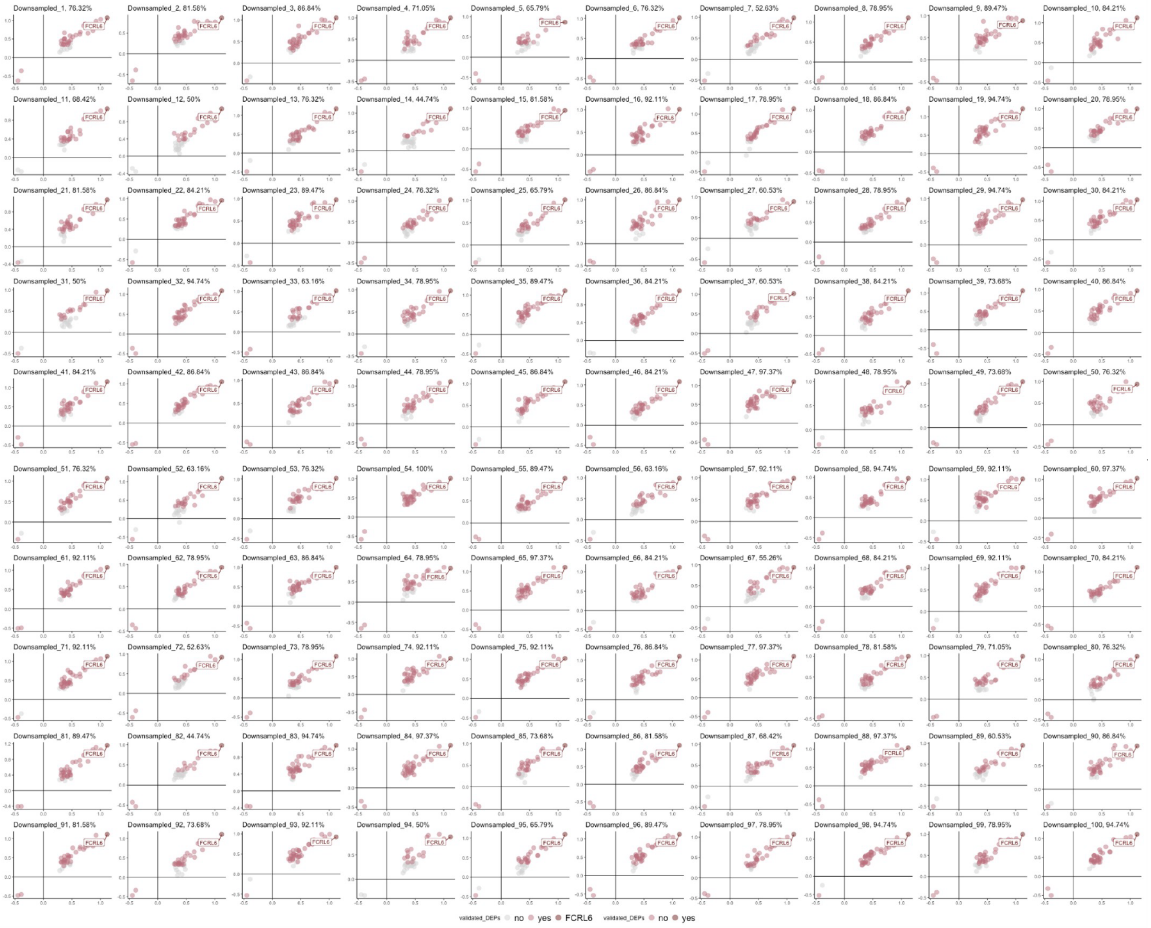

Supplementary Figure S5. Protein downsampling analysis.

The concordance plots for re-performing the proteomics analysis across 100 downsampling iterations in the discovery cohort. The percentages of significant proteins (FDR < 0.05) replicated in the downsampling iterations are in the range between 44.74% - 100.00 % with mean = 80.13%. The x-axis is the effect size from original analysis. The y-axis is the effect size from downsampling analysis.

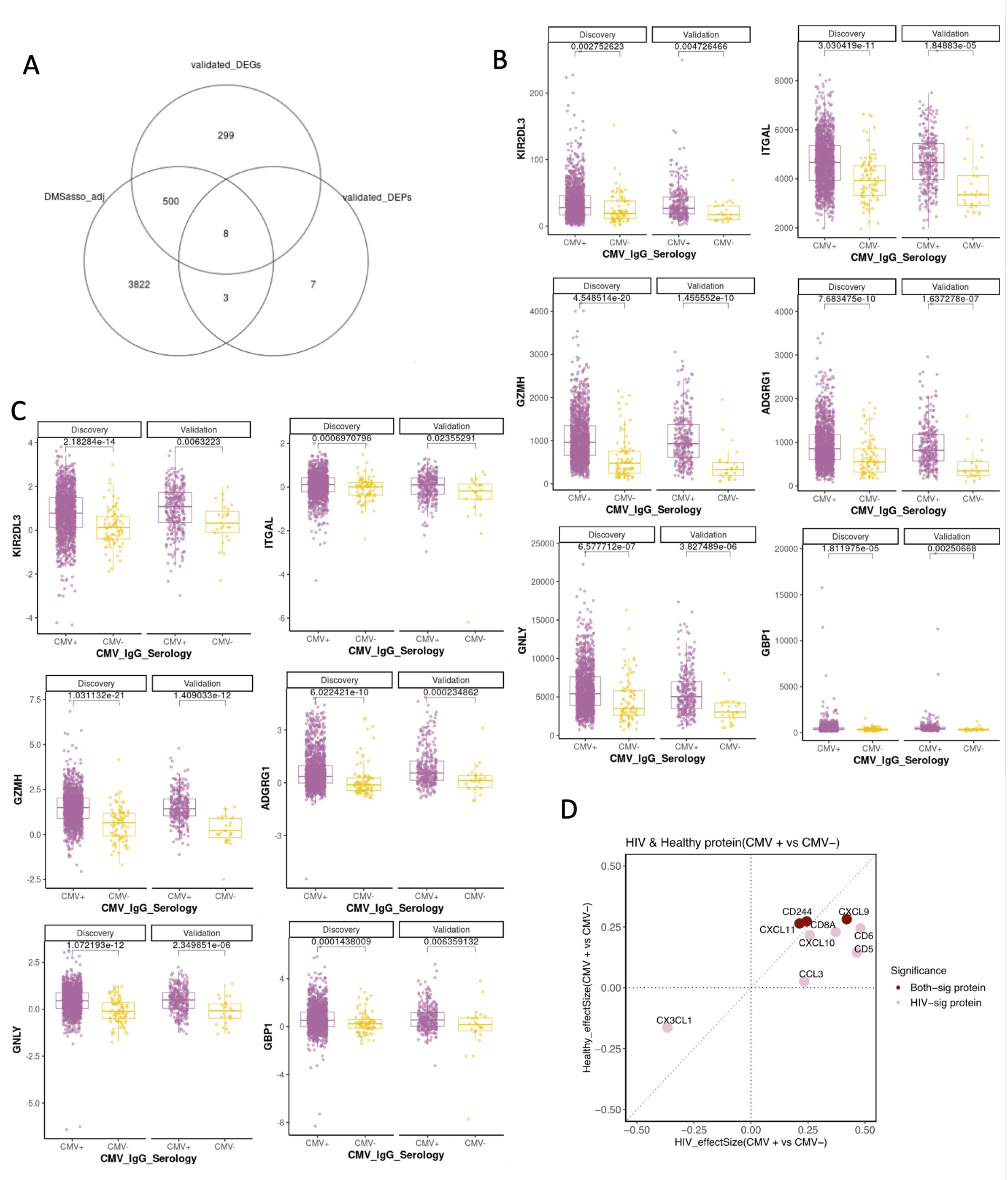

Supplementary Figure S6. Transcriptome and proteome analysis

A. Venn diagram between eQTM (labelled as DMSasso_adj, which were significant in both discovery and validation cohort), validated DEGs, and validated DEPs

B. The gene expression levels of 7 genes which were significantly different between CMV+/- in both gene expression and protein levels (as mentioned in Fig. 3D, except FCRL6)

C. The protein abundance of 7 proteins which were significantly different between CMV+/- in both gene expression and protein levels (as mentioned in Fig. 3D, except *FCRL6*)

D. Comparison of effect sizes between nominally significant proteins ($P < 0.05$) between CMV+ and CMV- in PLHIV and matching proteins in a healthy cohort.

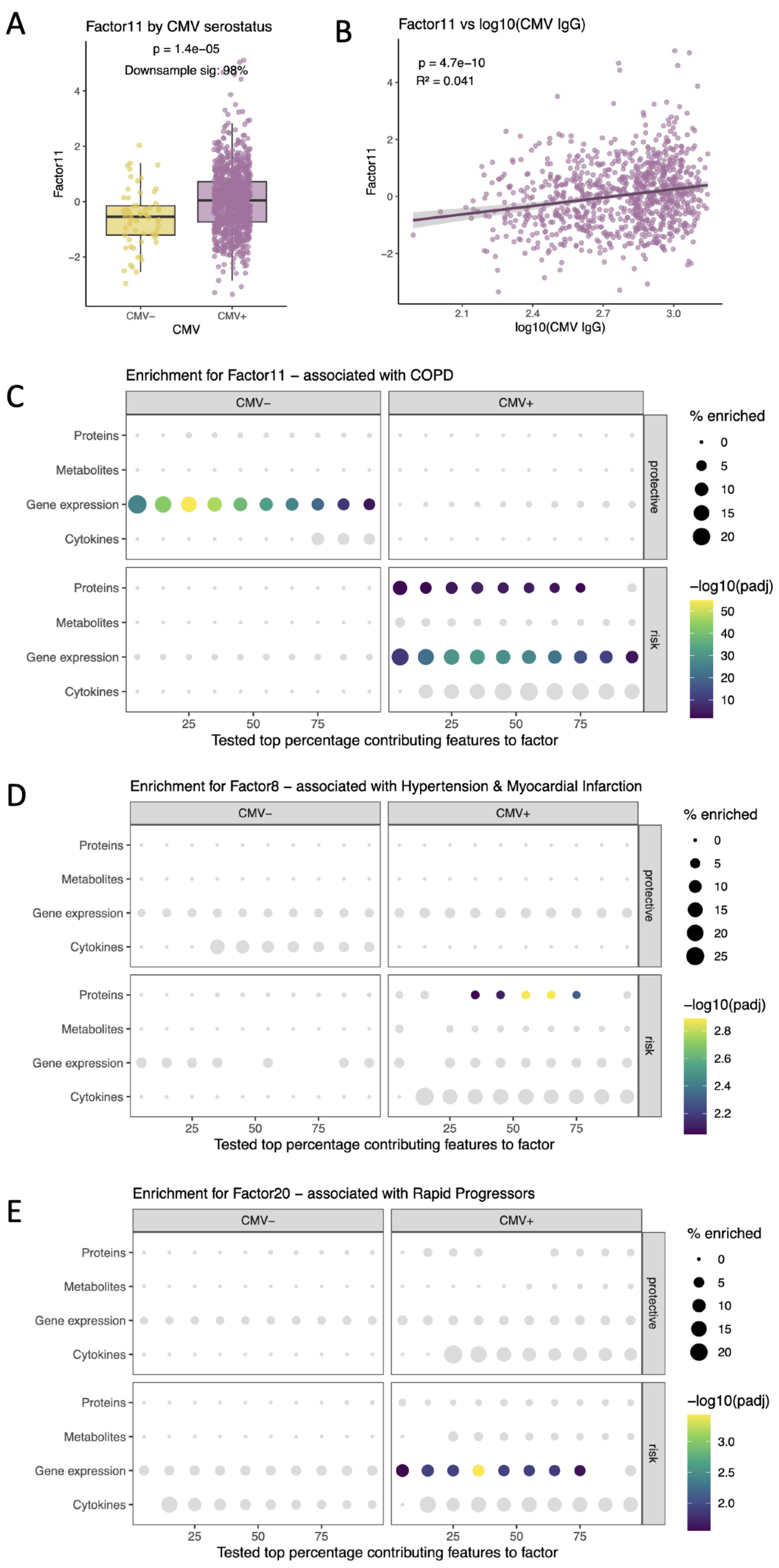

Supplementary Figure S7. Analysis of CMV-molecules and comorbidity-linked MOFA factors

A. Boxplot of CMV serostatus (x-axis) plotted against Factor 11 values (y-axis).
B. Scatterplot of CMV IgG titers (x-axis) plotted against Factor 11 values (y-axis). This plot includes only CMV+ individuals.

C. Enrichment analysis of Factor 11. We tested for enrichment iteratively from 5% until 95% of the top features for each LF. Only dots passing multiple testing corrections are colored (FDR < 0.05).

D. Enrichment analysis of Factor 8.

E. Enrichment analysis of Factor 20.

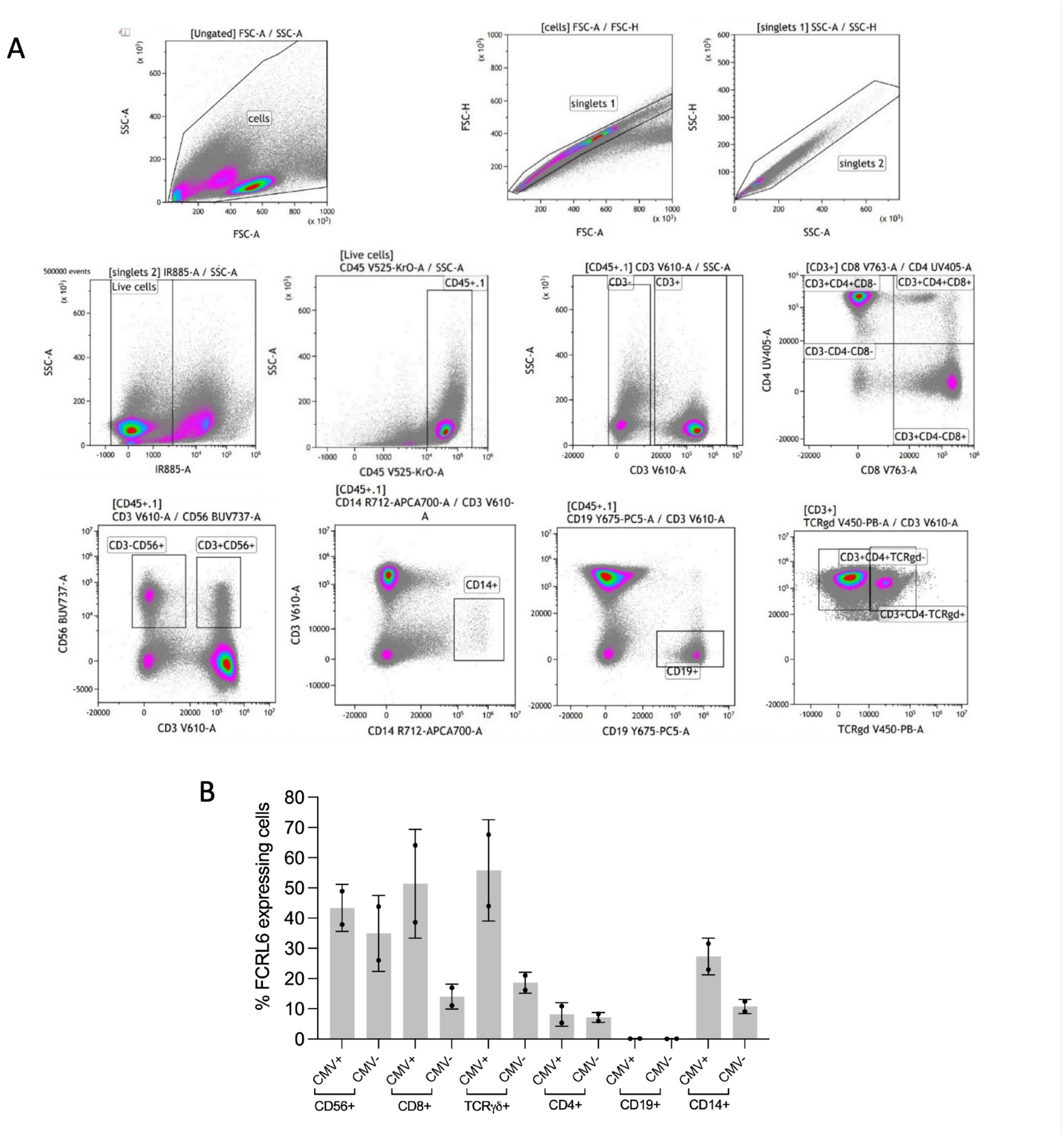

Supplementary Figure S8. Flow cytometry experiment FCRL6

A. Illustrating the sequential flow-cytometric gating strategy used to quantify *FCRL6*-expressing immune subsets in PBMCs from PLHIV

B. Bar graph showing the percentage of FCRL6+ cells within the different immune cell subsets. The data is stratified based on the CMV status, including 2 PLHIV CMV+ and 2 PLHIV CMV-.

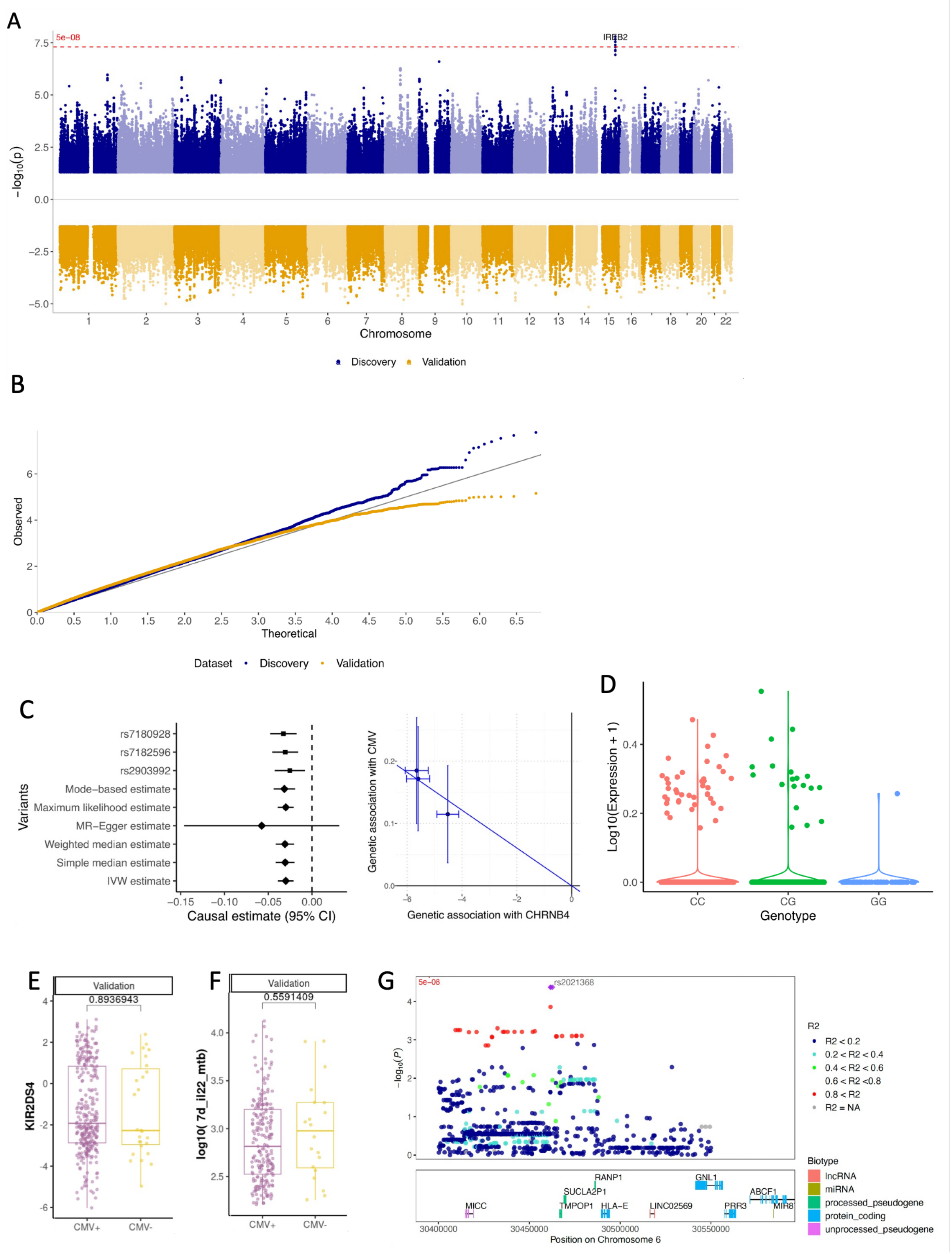

Supplementary Figure S9. Genetic analysis

A. Manhattan plot of CMV +/- (in European cohort). The y-axis is the p-value transformed by -log10, while the x-axis is the position of each SNP on the genome (GRCh38). The canonical 5e-8 was used as the genome-wide significant threshold which is indicated by the dashed red line. The color indicates the cohorts, including discovery (blue) and validation (orange)

B. Q-Q plot of GWAS results. The inflation parameter lambda are 1.08 and 0.96 for discovery and validation respectively.

C. MR analysis between *CHRNB4* expression and PLHIV resistance to CMV.

D. eQTL effect of *CHRNB4* at rs7180928 locus.

E. The abundance of KIR2DS4 in the validation cohort.

F. The abundance of IL22 stimulated by *M.* tb on the 7th day.

G. Locus plot of the HLA-E locus from the CMV GWAS.

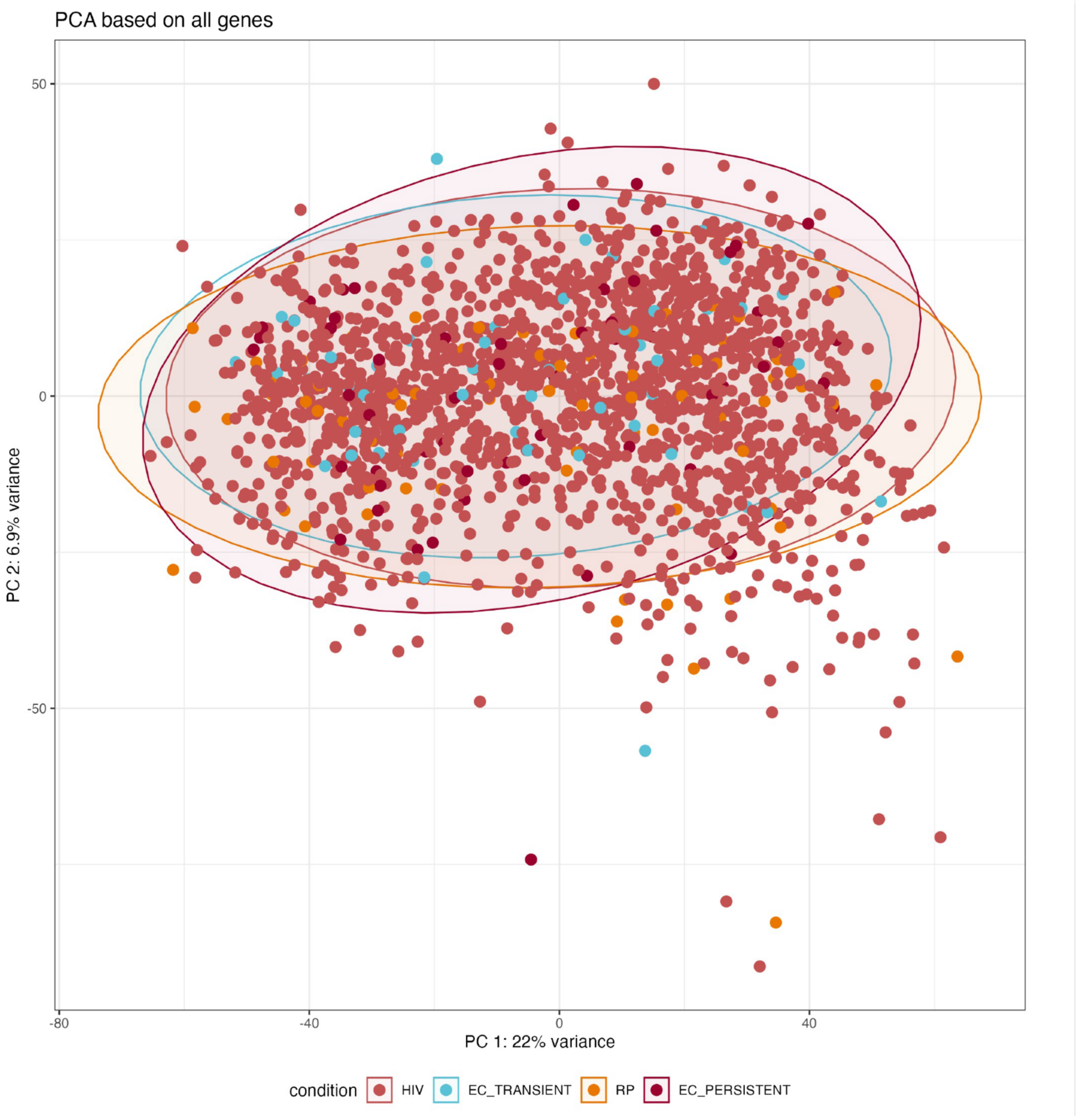

Supplementary Figure S10. PCA of RNAseq

PCA was performed on normalized expression values of all genes in the discovery cohort. The first principal component (PC1) explains 22% of the variance and the second principal component (PC2) explains 6.8% of the variance. Each point represents a single individual, color-coded by clinical condition: HIV-infected progressors (HIV; red), elite controllers with transient CMV reactivation (EC_TRANSIENT; cyan), rapid progressors (RP; orange), and elite controllers with persistent CMV reactivation (EC_PERSISTENT; dark red). Shaded ellipses denote the 95% confidence interval for each group.
