# Supplementary material for: Molecular signatures and causal factors underlying latent cytomegalovirus infection among people living with HIV (PLHIV)
Source: Nat Commun. 2026 Mar 25;17:2871. doi: 10.1038/s41467-026-70889-z (PMC13022287; doi:10.1038/s41467-026-70889-z)
Supplement: Supplementary file 3 — Description of Additional Supplementary Files [file 41467_2026_70889_MOESM3_ESM.pdf]

Supplementary Data

Supplementary Data S1: Sample size of the cohort and each omics dataset involved in present study

Supplementary Data S2: Cytokine production associations to CMV seropositivity in the discovery cohort

Supplementary Data S3: Cytokine production associations to CMV seropositivity in the validation cohort

Supplementary Data S4: Immune cell subset (absolute cell count) associations to CMV seropositivity in the discovery cohort

Supplementary Data S5: Immune cell subset (absolute cell count) associations to CMV seropositivity in the validation cohort

Supplementary Data S6: Gene expression (bulk RNAseq) associations to CMV seropositivity in the discovery cohort

Supplementary Data S7: Gene expression (bulk RNAseq) associations to CMV seropositivity in the validation cohort

Supplementary Data S8: Significant pathways (qvalue < 0.05) from pathway analysis using validated DEGs (sig. in both discovery and validation cohort)

Supplementary Data S9: Plasma protein abundance (Olink) associations to CMV seropositivity in the discovery cohort

Supplementary Data S10: Plasma protein abundance (Olink) associations to CMV seropositivity in the validation cohort

Supplementary Data S11: Plasma metabolite abundance associations to CMV seropositivity in the discovery cohort

Supplementary Data S12: Plasma metabolite abundance associations to CMV seropositivity in the validation cohort

Supplementary Data S13: DNA methylation associations to CMV seropositivity in the discovery cohort - Subset of significant (FDR<0.05) CpG sites located in FCRL6

Supplementary Data S14: DNA methylation associations to CMV seropositivity in the discovery cohort - Subset of significant (FDR<0.05) CpG sites located in non-classical HLA genes

Supplementary Data S15: Alleles at the MHC genetic region related to FCRL6 gene expression

Supplementary Data S16: Colocalization between identified GWAS locus with eQTL in the locus
