# Supplementary material for: Molecular signatures and causal factors underlying latent cytomegalovirus infection among people living with HIV (PLHIV)
Source: Nat Commun. 2026 Mar 25;17:2871. doi: 10.1038/s41467-026-70889-z (PMC13022287; doi:10.1038/s41467-026-70889-z)
Supplement: Supplementary file 5 — Reporting Summary [file 41467_2026_70889_MOESM5_ESM.pdf]

nature portfolio

Corresponding author(s): *Double-anonymous peer review submissions: write DAPR and your manuscript number here instead of author names.*

Last updated by author(s): *YYYY-MM-DD*

Reporting Summary

Nature Portfolio wishes to improve the reproducibility of the work that we publish. This form provides structure for consistency and transparency in reporting. For further information on Nature Portfolio policies, see our Editorial Policies and the Editorial Policy Checklist.

Statistics

For all statistical analyses, confirm that the following items are present in the figure legend, table legend, main text, or Methods section.

n/a	Confirmed	
☐	☒	The exact sample size (*n*) for each experimental group/condition, given as a discrete number and unit of measurement
☐	☒	A statement on whether measurements were taken from distinct samples or whether the same sample was measured repeatedly
☐	☒	The statistical test(s) used AND whether they are one- or two-sided<br>*Only common tests should be described solely by name; describe more complex techniques in the Methods section.*
☐	☒	A description of all covariates tested
☐	☒	A description of any assumptions or corrections, such as tests of normality and adjustment for multiple comparisons
☐	☒	A full description of the statistical parameters including central tendency (e.g. means) or other basic estimates (e.g. regression coefficient) AND variation (e.g. standard deviation) or associated estimates of uncertainty (e.g. confidence intervals)
☐	☒	For null hypothesis testing, the test statistic (e.g. *F*, *t*, *r*) with confidence intervals, effect sizes, degrees of freedom and *P* value noted<br>*Give P values as exact values whenever suitable.*
☒	☐	For Bayesian analysis, information on the choice of priors and Markov chain Monte Carlo settings
☒	☐	For hierarchical and complex designs, identification of the appropriate level for tests and full reporting of outcomes
☐	☒	Estimates of effect sizes (e.g. Cohen's *d*, Pearson's *r*), indicating how they were calculated

Our web collection on statistics for biologists contains articles on many of the points above.

Software and code

Policy information about availability of computer code

Data collection	Data collection involved the use of several commercial and open-source tools: • Flow Cytometry: CytoFLEX-LX software (Beckman Coulter, version 2.3) for immune cell profiling. • Proteomics: Olink® Explore software for processing data from the Olink Explore 3072 platform. • RNASequencing: STAR alignment software (version 2.7.9a) for RNA-seq data processing and alignment. • DNAMethylation: Minfi package in R (version 1.36) for processing methylation data from the Illumina platform.
Data analysis	Data analysis utilized a combination of custom code and open-source R packages: • RPackages: DESeq2 (version 1.30.0) for differential expression analysis, limma (version 3.46.0) for epigenome-wide association studies, and TwoSampleMR (version 0.5.6) for Mendelian randomization. • Custom R andPythonscripts were developed to manage data preprocessing and statistical analyses, available in a GitHub repository (https://github.com/CiiM-Bioinformatics-group/2000HIV_CMV). All code and scripts for data analysis are documented and accessible for reproducibility.

For manuscripts utilizing custom algorithms or software that are central to the research but not yet described in published literature, software must be made available to editors and reviewers. We strongly encourage code deposition in a community repository (e.g. GitHub). See the Nature Portfolio guidelines for submitting code & software for further information.

Data

Policy information about availability of data

All manuscripts must include a data availability statement. This statement should provide the following information, where applicable:

- Accession codes, unique identifiers, or web links for publicly available datasets
- A description of any restrictions on data availability
- For clinical datasets or third party data, please ensure that the statement adheres to our policy

The datasets generated and analyzed during the current study are available from the corresponding author on reasonable request. Due to the sensitive nature of health information from people living with HIV, data access is restricted to comply with privacy and ethical guidelines. For approved data sharing, anonymized data will be provided under a data use agreement. Omics data supporting the findings of this study will be deposited in both the Radboud Data Repository (RDR) and the European Genome-Phenome Archive (EGA), with accession codes available upon publication.

Research involving human participants, their data, or biological material

Policy information about studies with human participants or human data. See also policy information about sex, gender (identity/presentation), and sexual orientation and race, ethnicity and racism.

Reporting on sex and gender	The study included both biological sex and self-reported gender information. The cohort consisted predominantly of males, reflecting the demographics of PLHIV in the sample population. Sex and gender were considered as potential confounding factors and were adjusted for in statistical analyses. Consent was obtained for data sharing, and individual-level data were aggregated by sex where applicable. Sex- and gender-based analyses were not the primary focus of the study but were accounted for in adjustments.
Reporting on race, ethnicity, or other socially relevant groupings	Ethnicity was recorded to explore genetic and immune profile differences among participants. Categories were based on selfreported ancestry, and genetic data were used to control for population stratification in statistical models. Race and ethnicity were analyzed together to support multi-omics data interpretation, with a primary focus on European ancestry in the validation cohort to ensure data consistency
Population characteristics	The study population was PLHIV with a mean age of 51.6 years, predominantly male, and diverse in genetic backgrounds. Participants were on stable antiretroviral therapy (ART) and were stratified based on CMV serostatus (seropositive and seronegative). Cohort characteristics, including age, BMI, and ethnicity, were factored into the statistical analyses to control for confounding effects.
Recruitment	Participants were recruited from Dutch HIV treatment centers, specifically targeting individuals with stable HIV management on ART. Recruitment strategies were designed to minimize self-selection bias, although the study may inherently reflect treatment-seeking individuals with access to healthcare.
Ethics oversight	The study was approved by the ethics committees of participating Dutch medical institutions. All participants provided informed consent for participation and data sharing in accordance with institutional and national guidelines

Note that full information on the approval of the study protocol must also be provided in the manuscript.

Field-specific reporting

Please select the one below that is the best fit for your research. If you are not sure, read the appropriate sections before making your selection.

[x] Life sciences [ ] Behavioural & social sciences [ ] Ecological, evolutionary & environmental sciences

For a reference copy of the document with all sections, see nature.com/documents/nr-reporting-summary-flat.pdf

Life sciences study design

All studies must disclose on these points even when the disclosure is negative.

Sample size	The sample size of 1887 participants was determined based on the availability of PLHIV with known CMV serostatus in the 2000HIV study cohort. No formal sample size calculation was conducted. The cohort size was deemed sufficient to observe statistically significant associations across multi-omics layers (e.g., DNA methylation, proteomics, and transcriptomics) between CMV seropositive and seronegative groups.
Data exclusions	Data exclusions were made for samples that failed quality control criteria. These included samples with low call rates in DNA methylation, poor RNA-seq alignment, and outliers in proteomic data as identified by PCA. Exclusion criteria were pre-established to ensure data integrity and reproducibility across multi-omics layers.
Replication	The study design included a validation cohort to replicate key findings from the discovery cohort. All significant associations in the discovery cohort (e.g., methylation and gene expression changes associated with CMV seropositivity) were tested and confirmed in the independent validation cohort, ensuring reproducibility of the results.
Randomization	Participants were not randomly allocated to experimental groups as the study is observational and based on the naturally occurring CMV serostatus of PLHIV. However, confounding factors such as age, sex, and BMI were controlled for in statistical models to adjust for nonApril

	2023 2randomized allocation.
Blinding	Investigators were not blinded to CMV serostatus during data analysis as this information was integral to the study's aim of comparing CMV seropositive and seronegative groups.

Reporting for specific materials, systems and methods

We require information from authors about some types of materials, experimental systems and methods used in many studies. Here, indicate whether each material, system or method listed is relevant to your study. If you are not sure if a list item applies to your research, read the appropriate section before selecting a response.

Materials & experimental systems

n/a	Involved in the study
☐	☒ Antibodies
☒	☐ Eukaryotic cell lines
☒	☐ Palaeontology and archaeology
☒	☐ Animals and other organisms
☐	☒ Clinical data
☒	☐ Dual use research of concern
☒	☐ Plants

Methods

n/a	Involved in the study
☒	☐ ChIP-seq
☐	☒ Flow cytometry
☒	☐ MRI-based neuroimaging

Antibodies

Antibodies used	The following fluorochrome-conjugated antibodies and dyes were used for flow cytometry of human PBMCs:<br>• LIVE/DEAD Fixable Viability Dye, ViaKrome (Beckman Coulter, Brea, CA, USA)<br>• CD3 (BV605, BioLegend, San Diego, CA, USA)<br>• CD4 (BUV395, BioLegend)<br>• CD8 (BV785, BioLegend)<br>• CD14 (AF700, BioLegend)<br>• CD19 (PE-Cy5, BioLegend)<br>• CD45 (Krome Orange, Beckman Coulter, Brea, CA, USA)<br>• CD56 (BUV737, BioLegend)<br>• TCRγδ (BV421, BioLegend)<br>• FCRL6 (APC, BioLegend)
Validation	All antibodies listed above are commercially validated for human PBMCs and for flow cytometry by their manufacturers.

Clinical data

Policy information about clinical studies

All manuscripts should comply with the ICMJE guidelines for publication of clinical research and a completed CONSORT checklist must be included with all submissions.

Clinical trial registration	The 2000HIV study is registered with ClinicalTrials.gov under the identifier NCT03994835.
Study protocol	The study protocol for the 2000HIV study was reviewed and approved by the Independent Review Board Nijmegen (IRB number: NL68056.091.81) and is accessible on clinicaltrials.gov. The 2000HIV study has also been previously described at https://doi.org/10.3389/fimmu.2022.982746.
Data collection	Data were collected across four Dutch medical centers from October 2019 to October 2021. Additional data on COVID-19 were also collected for participants recruited after the start of the COVID-19 pandemic. Written informed consent was received from participants prior to inclusion in the study. All experiments with human samples were conducted according to the principles expressed in the Declaration of Helsinki.
Outcomes	The outcomes of the 2000HIV study focus on uncovering biological mechanisms that influence variability in immune responses within the human population, aiming specifically to identify pathways and biomarkers associated with persistent inflammation and accelerated aging, non-AIDS-related comorbidities, extreme clinical phenotypes, and the HIV reservoir.

Plants

Seed stocks	*Report on the source of all seed stocks or other plant material used. If applicable, state the seed stock centre and catalogue number. If plant specimens were collected from the field, describe the collection location, date and sampling procedures.*
Novel plant genotypes	*Describe the methods by which all novel plant genotypes were produced. This includes those generated by transgenic approaches, gene editing, chemical/radiation-based mutagenesis and hybridization. For transgenic lines, describe the transformation method, the number of independent lines analyzed and the generation upon which experiments were performed. For gene-edited lines, describe the editor used, the endogenous sequence targeted for editing, the targeting guide RNA sequence (if applicable) and how the editor was applied.*
Authentication	*Describe any authentication procedures for each seed stock used or novel genotype generated. Describe any experiments used to assess the effect of a mutation and, where applicable, how potential secondary effects (e.g. second site T-DNA insertions, mosiacism, off-target gene editing) were examined.*

Flow Cytometry

Plots

Confirm that:

- [x] The axis labels state the marker and fluorochrome used (e.g. CD4-FITC).
- [x] The axis scales are clearly visible. Include numbers along axes only for bottom left plot of group (a 'group' is an analysis of identical markers).
- [x] All plots are contour plots with outliers or pseudocolor plots.
- [x] A numerical value for number of cells or percentage (with statistics) is provided.

Methodology

Sample preparation	peripheral blood mononuclear cells (PBMCs) of people living with HIV
Instrument	CytoFLEX flow cytometer (Beckman Coulter, Brea, CA, USA)
Software	Kaluza 2.3.0 (Beckman Coulter, Brea, CA, USA).
Cell population abundance	The cell population of interest were determined upon staining with following above-mentioned antibodies/dyes.<br><br>The abundance:<br>- CD56+ NK cells, mean 43.3% in CMV+ and 34.9% in CMV-<br>- CD8+ T cells, mean 51.3% in CMV+ and 14.01% in CMV-<br>- TCRγδ+ T cells, mean 55.8% in CMV+ and 18.6% in CMV-<br>- CD4+T cells, mean 8.1% in CMV+ and 7.1% in CMV-<br>- CD19+ B cells, mean 0.14% in CMV+ and 0.06% in CMV-<br>- CD14+ monocytes, mean 27.2% in CMV+ and 10.7% in CMV-
Gating strategy	All cells: defined by FSC-A (x-axis) vs SSC-A (y-axis). Singlets: SSC-H (y-axis) vs SSC-A (x-axis). Live cells: Viakrome– population gated from singlets. Leukocytes: CD45+ cells within Live gate. Total CD3+ T cells: CD3+ cells within Live/CD45+. CD4+ T cells: CD3+CD4+CD8– cells within Live/CD45+. CD8+ T cells: CD3+CD8+CD4– cells within Live/CD45+. NK cells: CD56+ cells within Live/CD45+. Monocytes: CD3–CD14+ cells within Live/CD45+. B cells: CD3–CD19+ cells within Live/CD45+. TCR T cells: CD3 +TCR+ cells within Live/CD45+.For FcRL6 expression in the mentioned cell types, a fluorescence minus one (FMO) control was included to define the positive populations.

- [x] Tick this box to confirm that a figure exemplifying the gating strategy is provided in the Supplementary Information.
